# Impact of deoxygenation/reoxygenation processes on the superconducting properties of commercial coated conductors

**DOI:** 10.1038/s41598-023-44086-7

**Published:** 2023-10-07

**Authors:** Pablo Cayado, Marco Bonura, Celia Lucas, Enora Saule, Hannes Rijckaert, Tommaso Bagni, Konstantina Konstantopoulou, Matteo Alessandrini, Carmine Senatore

**Affiliations:** 1 Department of Quantum Matter Physics, University of Geneva, Geneva, Switzerland; 2 Department of Chemistry, Ghent University, Krijgslaan 281-S3, 9000 Ghent, Belgium; 3 Bruker BioSpin, Industriestrasse 26, 8117 Fällanden, Switzerland; 4 Department of Nuclear and Particle Physics, University of Geneva, Geneva, Switzerland

**Keywords:** Condensed-matter physics, Superconducting properties and materials

## Abstract

We report the evolution of the superconducting properties of a commercial coated conductor during deoxygenation and reoxygenation processes. By analyzing the changes on the critical temperature, *T*_c_, and critical current density, *J*_c_, at 4 and 77 K, we have identified the conditions that cause a complete deoxygenation of the coated conductor and, also, the reoxygenation conditions that allow a recovery of the superconducting properties. A complete suppression of superconductivity happens at ~ 500–550 °C under a pure argon flow. After a complete deoxygenation, we observed that a reoxygenation process at ~ 400–450 °C in pure oxygen flow allows, not only a full recovery, but even an improvement in *J*_c_, both at 4 and 77 K. Such an increase of *J*_c_ is kept or even enhanced, especially at 77 K, in the presence of magnetic fields up to ~ 6 T. A microstructural analysis by transmission electron microscopy did not give evidence of major differences in the densities of Y_2_O_3_ nanoparticles and stacking faults between the pristine and reoxygenated samples, suggesting that these defects should not be the cause of the observed enhancement of *J*_*c*_. Therefore, the combined action of other types of defects, which could appear as a consequence of our reoxygenation process, and of a new level of oxygen doping should be responsible of the *J*_c_ enhancement. The higher *J*_c_ that can be achieved by using our simple reoxygenation process opens new parameter space for CCs optimization, which means choosing a proper *p*O_2_-temperature–time trajectory for optimizing *J*_c_.

## Introduction

In recent years, the industrial production of high-performance second-generation superconducting tapes or Coated Conductors (CCs) has drastically increased. This allows envisaging a near future in which the use of CCs in the fabrication of devices such as high-field magnets, fault-current limiters or motors will be a reality on a routine basis^1,2^. The production of CCs is a complex process. It starts by depositing several buffer layers on top of a metallic substrate serving as chemical protection and seed for the growth of the superconducting layer that is made of *RE*Ba_2_Cu_3_O_7-δ_ (*RE*BCO)^3,4^. Then, the *RE*BCO layer is coated with silver (Ag) and copper as protective and thermal stabilizer layers, respectively.

There are many difficulties that can appear during the preparation of this intricate multilayer structure, but most of them are related with the *RE*BCO layer. It needs to be grown epitaxially to minimize the harmful effects of the grain boundaries that can drastically reduce the superconducting current flow^5,6^. Furthermore, the oxygen content in the superconducting layer must be carefully controlled to induce the needed transition from the tetragonal non-superconducting phase to the orthorhombic superconducting one. Achieving the proper oxygen content is crucial to maximize the performances of the CCs and, therefore, understanding and optimizing the oxygenation process is a very relevant topic. This process depends on the kinetics of the oxygen diffusion processes, oxygen incorporation (in-diffusion) and excorporation (out-diffusion), that take place in the *RE*BCO superconducting layer. Therefore, an improved comprehension of these diffusion processes in *RE*BCO films is needed to design an optimal oxygenation during the CCs fabrication.

The oxygen diffusion in *RE*BCO films has been extensively studied in the past, especially in films deposited on single crystals. Moreover, the vast majority of the articles focused on the mechanism and the kinetics of the process by doing measurements with techniques like secondary ion mass spectrometry^7–9^, oxygen tracer^10^, thermogravimetry^11^, spectroscopic ellipsometry^12^ or in-situ conductance^13–21^. All of these studies demonstrate that comprehending and controlling the diffusion process is essential to properly oxygenate the *RE*BCO films and maximize their performance. Furthermore, they provide evidence that variations in the microstructure among films deposited on single crystals using different techniques lead to distinct kinetics of the oxygen diffusion in each case. It is important to note that due to the dissimilar microstructures between films deposited on single crystals and CCs—the latter having typically a larger amount of low-angle grain boundaries and pores compared to films deposited on single crystals using the same techniques–, results from experiments involving films on single crystals cannot be directly extrapolated to the CCs.

However, only a minor group of works deal with the issue of the oxygenation of CCs and, even less, concentrate in the superconducting properties, particularly on the critical current density (*J*_c_). Within this group, most of the articles report on the problem of the damages that heating can cause on them^22–25^. These reports clarify that the exposure of CCs to heat, either as a consequence of their use for manufacturing different devices (soldering, impregnation curing, etc.) or by accident, e.g., in a magnet quench, produce a degradation of the superconducting properties due to the oxygen out-diffusion. Especially relevant is the work carried out in our group by Bonura et al*.* that describes in detail the reasons why even at temperatures as low as ~ 170 °C there can be an evident decrease of the critical current. This “low temperature” degradation is linked to the oxygen diffusion process through the grain boundaries, a process that is activated at much lower temperatures than the out-diffusion from the interior of the grains^22^.

Presently, there is not much information about the conditions to restore the oxygen content on a partially or completely deoxygenated CC, i.e., how a reoxygenation process can restore the superconducting properties of a CC. In fact, to the best of our knowledge, most of the studied on this topic have been carried out on *RE*BCO deposited on single crystal substrates^19^. Only the work of Kim et al*.* reports about this issue on CCs, but on narrow ranges of deoxygenation and oxygenation temperatures^26^. The clear differences between the *RE*BCO films deposited on a single crystal substrate or on a buffered metallic tape (thickness, grain orientation, formation of grain boundaries, presence of protective layers, etc.) mean that the conclusions that one can get from the first could not be applied straightforwardly to the second.

The present work tries to fill the lack of information on this topic presenting a comprehensive study of the deoxygenation and oxygenation processes in a commercial CC. The aim was to find out if, after a complete deoxygenation of a coated conductor, it is possible to restore its superconducting properties by doing a simple reoxygenation process. By means of magnetic, microstructural, and transport measurements we provide insights into i) the conditions that lead to a partial or complete deoxygenation of the CC, and ii) how to reestablish a significant oxygen doping in the CC that allow to obtain good values of critical temperature and critical current density through reoxygenation processes.

## Methods

### Preparation of the samples

The commercial coated conductor employed in this work was produced by SuperOx using YBa_2_Cu_3_O_7-δ_ (YBCO) as superconducting layer. The tape is 12 mm-wide and the YBCO layer is ~ 3 µm thick, according to the manufacturer specifications. The superconductor is protected by a ~ 2 µm thick Ag layer; a ~ 1 µm-thick layer of Ag coats also the substrate side. The nominal critical current, *I*_c_, at 77 K in self-field is 595 A. No copper stabilizer is present in this tape. The architecture and deposition techniques of the different CC layers are described in detail in reference^1^. Small pieces of ~ 2.5 × 2.5 mm^2^ or larger ones of 90 × 4 mm^2^ were cut from the CC by scissors. The use of scissors to cut the samples can create a certain damage, especially in the smallest samples. We estimated this deterioration by measuring inductively the nominal self-field critical current density ($J_{{\text{c}}}^{{{\text{sf}}}}$) at 4 K of ~ 20 pieces of ~ 2.5 × 2.5 mm^2^, finding maximum differences between them of ~ 20%. For the experiments presented in this paper, the Ag protective layer was removed in the small samples using a 1:1 mixture (in volume) of ammonia and 30% hydrogen peroxide. We evaluated also the impact of the Ag etching on the nominal $J_{{\text{c}}}^{{{\text{sf}}}}$ by performing magnetic measurements on samples before and directly after etching and found a decrease of ~ 10% in $J_{{\text{c}}}^{{{\text{sf}}}}$ after the etching process.

### Thermal processes

**Figure 1:**
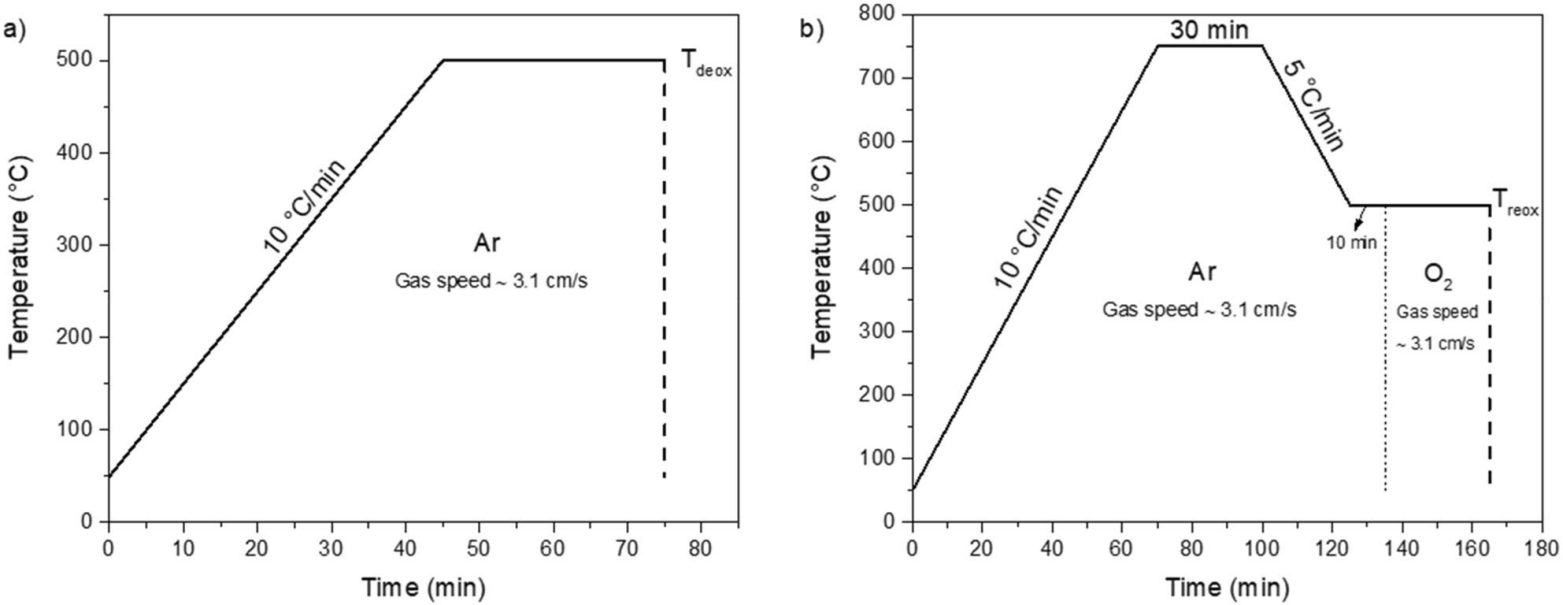
Examples of the thermal profiles followed to carry out (**a**) the deoxygenation and (**b**) the reoxygenation experiments.

Thermal treatments were carried out in tubular furnaces with a tube diameter of 2.6 cm. To study the deoxygenation process, the as-received samples were heated at 10 °C/min under pure argon (Ar) flow (residual oxygen ~ 1.5 ppm measured with an oxygen analyzer from *Michell Instruments*, gas speed ~ 3.1 cm/s) following the profile schematically shown in Fig. 1a. The maximum temperature (*T*_deox_) was changed in steps of 50 °C from 150 to 700 °C. Samples were kept at *T*_deox_ for 30 min and then quenched by moving the tube out of the heating zone of the furnace, which gives rather modest cooling rates estimated as ~ 300 °C/min in the first 300–400 °C of temperature decay.

The reoxygenation process was carried out following the profile displayed in Fig. 1b. First, the samples were heated in Ar up to 750 °C and this temperature was kept for 30 min. After this, the samples were cooled until the desired temperature *T*_reox_ was reached and stabilized. In this moment, the Ar flow was changed to O_2_ (gas speed ~ 3.1 cm/s) to reoxygenate the samples at *T*_reox_ for 30 min. *T*_reox_ was changed in steps of 50 °C from 150 °C to 700 °C. After the 30 min, the atmosphere was changed again to Ar and the samples were quenched. By changing the atmosphere from O_2_ to Ar during the quench we were trying to limit the change in the oxygen content during cooling, as the out-diffusion induced by Ar is much slower than the in-diffusion^19,21^, and considering the high cooling rates during the quench, the final oxygen content of the films is presumed to correspond to the one at the temperature at which the quench started. After completing the thermal process displayed in Fig. 1b, we say that a sample passed through a complete deoxygenation/reoxygenation process.

### Characterization of the samples

The field and temperature dependences of the magnetic moment were measured in the 2.5 × 2.5 mm^2^ samples to determine the critical temperature (*T*_c_) and critical current density (*J*_c_) of the samples in a SQUID-VSM magnetometer from *Quantum Design*. To measure *T*_c_, the samples were cooled down to ~ 10 K in zero-field and then a perpendicular (to the sample surface, parallel to the *c*-axis direction) field of ~ 10 Oe was applied. The magnetic moment of the sample was acquired while increasing the temperature from 10 to 100 K with a heating ramp rate of 1 K/min. The *T*_c_ values reported in this work correspond to the onset critical temperature, *T*_c_^onset^, which is extracted from the *m*(*T*) curves as reported in reference^22^. *J*_c_ was calculated from the field dependence of the magnetic moment, *m*(*B*), which was measured between 0 and 7 T at 4.2 K and 77 K, using the Bean’s critical state formula for a slab in perpendicular field^27,28^. When we refer to self-field values, we mean the maximum *J*_c_ that usually is reached for *B* = 0 T at 77 K and at low fields in the range of 0.1–0.2 T at 4 K due to the underlying granular nature of the samples that were used^22^.

All samples prepared in this work passed through a complete deoxygenation/reoxygenation cycle. We started with 12 pieces of as-received samples (size ~ 2.5 × 2.5 mm^2^) from which we removed the Ag coating by chemical etching. Pristine samples (“pristine” refers to the sample after the Ag etching) are first deoxygenated at a certain maximum deoxygenation temperature (*T*_deox_), following the thermal treatment shown in Fig. 1a, and then reoxygenated at a certain reoxygenation temperature (*T*_reox_), using the thermal treatment displayed in Fig. 1b. For a given sample, the thermal treatments are chosen in such a way that *T*_deox_ = *T*_reox_. *T*_c_ and *J*_c_ values measured on each of the 12 pristine samples were used as references to evaluate the relative variations after the corresponding deoxygenation and reoxygenation heat treatments. This allowed us to rule out spurious effects due to external factors as possible damage due to the sample cutting.

The microstructural properties were studied by scanning transmission electron microscopy (STEM, JEOL JEM-2000FS operated at 200 kV) with bright field (BF) detector. Cross-sectional STEM lamellas were prepared by ion milling via a focused ion beam in-situ lift-out procedure.

*I*_c_ at 77 K in self-field was obtained by transport measurements carried out in a liquid nitrogen bath on 90 mm long and 4 mm wide tape pieces. The Ag layer was not etched in this samples both for protection purposes and to facilitate the fixation of the voltage taps. The original 12 mm wide tape was cut longitudinally in 4 mm wide pieces to reduce *I*_c_ and allow an easier measurement without overcoming the limit of our current source (250 A). We adopted the standard 1 µV/cm criterion to evaluate *I*_c_ from the electric field vs. applied current curves.

## Results

### Deoxygenation process

**Figure 2:**
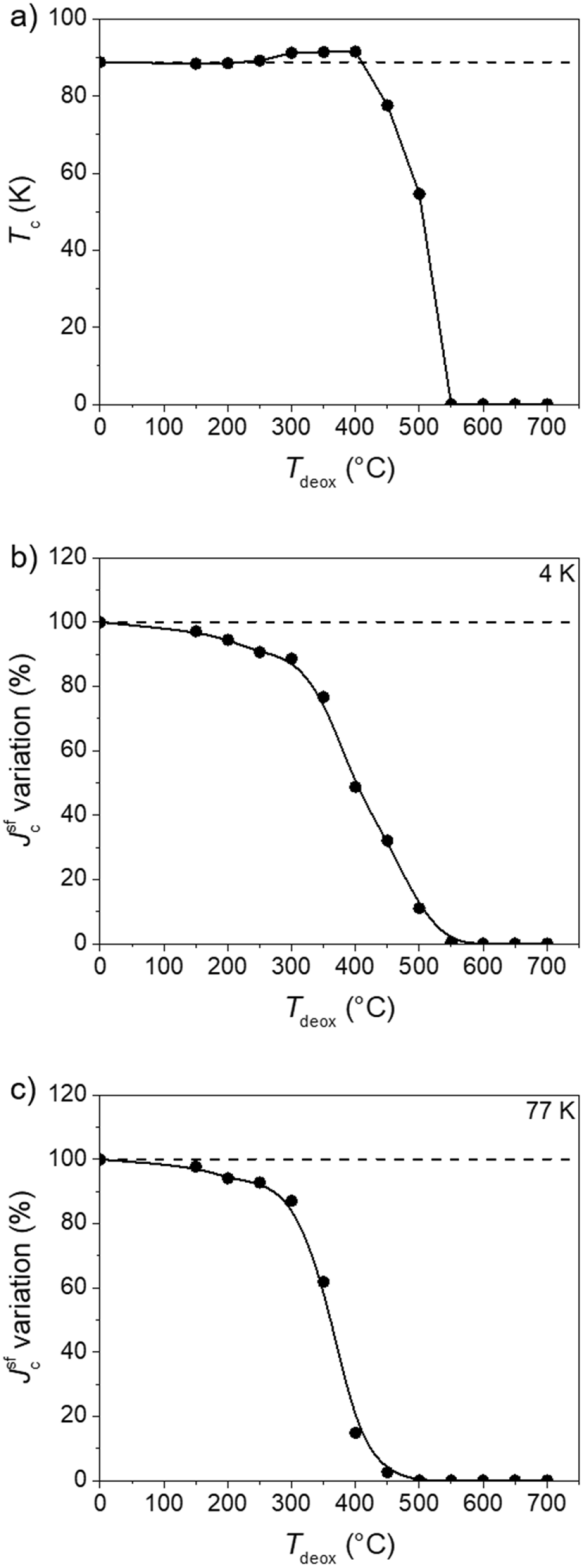
(**a**) *T*_c_ and self-field *J*_c_ ($J_{{\text{c}}}^{{{\text{sf}}}}$) variation at (**b**) 4 K and at (**c**) 77 K in dependence of the maximum temperature of deoxygenation (*T*_deox_) for samples deoxygenated during 30 min under pure Ar flow. The black horizontal dashed lines indicate the values of the pristine samples. The lines are only a guide for the eyes.

Figure 2 shows the dependence of (a) *T*_c_, (b) self-field critical current density ($J_{{\text{c}}}^{{{\text{sf}}}}$) at 4 K and (c) at 77 K on the maximum temperature (*T*_deox_) for different samples annealed during 30 min at different *T*_deox_.

*T*_c_ remains almost unchanged (~ 88–89 K) up to *T*_deox_ ~ 200 °C. For *T*_deox_ ≥ 250 °C, *T*_c_ starts to increase reaching a maximum of ~ 92.5 K at *T*_deox_ ~ 350–400 °C. For *T*_deox_ ≥ 450 °C there is a sudden decrease of *T*_c_. For *T*_deox_ ≥ 550 °C, no superconducting transition is observed in the investigated range of temperatures (10–100 K). As a convention, we attributed to these samples a *T*_c_ of 0 K. The observed evolution of *T*_c_ indicates that the samples are initially in an overdoped state. Indeed, the investigated CCs are intentionally overdoped by the manufacturer, with the scope of enhancing *J*_c_ at high fields in the 4–20 K range^1^. When an overdoped sample is heated, it loses oxygen and passes from the overdoped state to the optimally-doped one (in our particular case, this happens after a plateau at ~ 350 °C of 30 min) and then to the underdoped state at higher temperatures, following the well-known “*T*_c_ parabola”^29^.

The results presented in Fig. 2b show that for samples heated during 30 min, $J_{{\text{c}}}^{{{\text{sf}}}}$ at 4 K is continuously decreasing even at very low temperatures when *T*_c_ still remains unchanged. For example, at 150 °C the $J_{{\text{c}}}^{{{\text{sf}}}}$ value already decreased by ~ 3% and at 200 °C by ~ 5%. Above 450 °C there is a sudden decay of the $J_{{\text{c}}}^{{{\text{sf}}}}$. At 77 K the situation is very similar and, in fact, the shape of the curves is almost the same. The results clearly indicate that the samples are not anymore superconducting when doing a deoxygenation process at around 500–550 °C.

### Reoxygenation process

**Figure 3:**
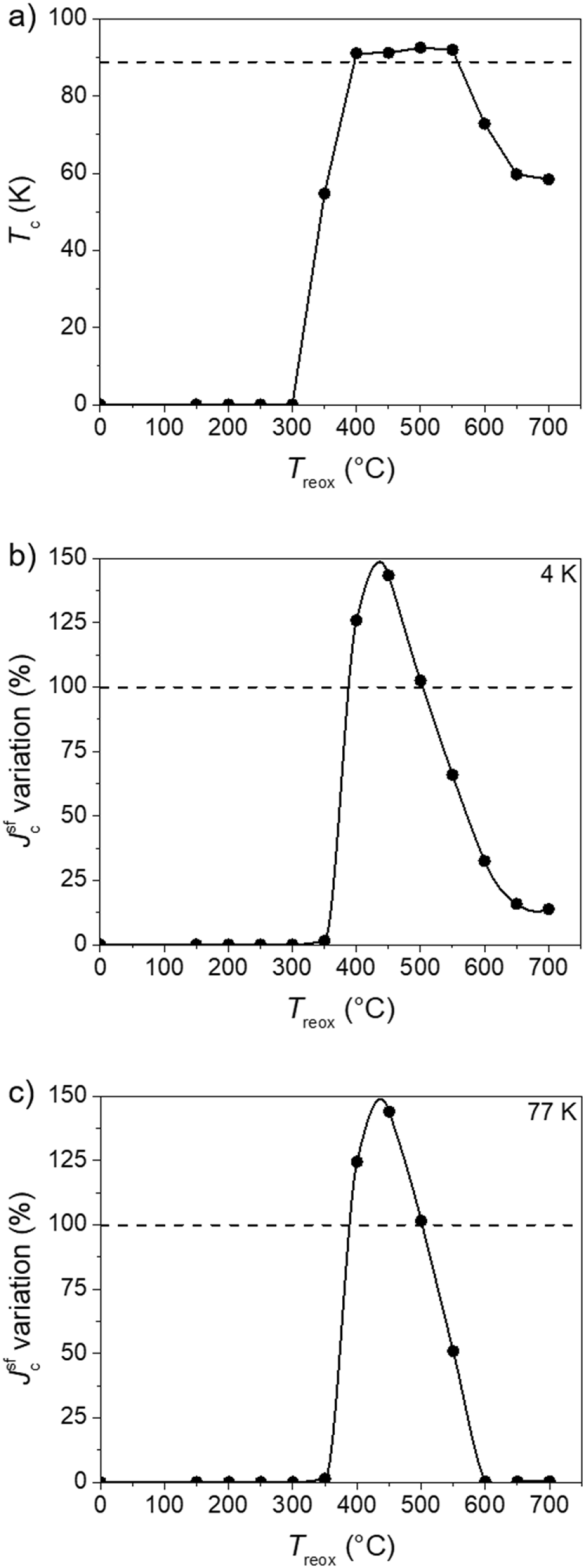
(**a**) *T*_c_ and self-field *J*_c_ ($J_{{\text{c}}}^{{{\text{sf}}}}$) variation at (**b**) 4 K and at (**c**) 77 K in dependence of the reoxygenation temperature (*T*_reox_) for samples reoxygenated during 30 min under pure O_2_ flow. The black horizontal dashed lines indicate the values of the pristine samples. The lines are only a guide for the eyes.

After the samples were deoxygenated following the thermal profile shown in Fig. 1a, the same samples were reoxygenated using the thermal profile displayed in Fig. 1b. Figure 3 presents the dependence of (a) *T*_c_, (b) self-field critical current density ($J_{{\text{c}}}^{{{\text{sf}}}}$) at 4 K and (c) at 77 K on the reoxygenation temperature (*T*_reox_) for samples reoxygenated during 30 min.

We observe that below 300 °C, *T*_c_ remains constant with no appreciable change. At these low temperatures, the oxygen in-diffusion is so slow that we do not observe any remarkable changes in the oxygen content inside the YBCO grains after 30 min of reoxygenation. Above 300 °C, the values of *T*_c_ suffer a strong variation. The in-diffusion is faster at these temperatures resulting in an increase of the oxygen content that, as expected, leads to larger values of *T*_c_. The oxygen content and, therefore, *T*_c_ is determined by the temperature and oxygen partial pressure at which the oxygenation is done^30^. According to the phase diagram, the largest oxygen contents are achieved at low temperatures^31^, but one should consider that the lower is the temperature, the slower is the in-diffusion. Therefore, at these low temperatures, it is necessary to find an equilibrium between the temperature and the time needed to maximize the oxygen content and thus *T*_c_. The *T*_c_ values arrive at a saturation at 450 °C when *T*_c_ enters in a plateau with values stable at ~ 91–92 K. It is noticeable that these values are not the same of the pristine samples, meaning that the oxygen content is different after the reoxygenation process. This plateau extends until 550 °C and then *T*_c_ starts decreasing. At these temperatures, the oxygen content accepted in the YBCO structure is lower so, it is expected that *T*_c_ decreases^31^. This reduction of *T*_c_ is quite drastic from 550 °C to 650 °C. Then the values tend to stabilize again suggesting the formation of a second “pseudo-plateau” in which *T*_c_ values are ~ 60 K. These values are in accordance with the data reported in reference^21^. According to this publication, the origin of this second plateau is the particular oxygen ordering in the Cu–O chains at these temperatures. In particular, the oxygen atoms are placed in the so-called Ortho-II structure where only half of the Cu–O chains are filled with oxygen following a sequence full-empty-full-empty-etc. This ordering is different from the one found in the first plateau that we observe at higher *T*_c_ of ~ 92 K where the oxygen atoms are placed in the so-called Ortho-I structure in which all the Cu–O chains are full of oxygen following the sequence full-full-full-etc^32–34^.

Similar to *T*_c_, $J_{{\text{c}}}^{{{\text{sf}}}}$ remains zero up to 300 °C. From this value of *T*_reox_ on, $J_{{\text{c}}}^{{{\text{sf}}}}$ starts increasing, reaches a maximum in the range 400–450 °C, and decreases above 450 °C. At 4 K the values do not reach zero at high *T*_reox_ although they fall below 25% of the values of the pristine samples. At 77 K, $J_{{\text{c}}}^{{{\text{sf}}}}$ drops to zero at 600 °C, as expected considering the strong decrease of *T*_c_. In the range 400–450 °C, we notice significant $J_{{\text{c}}}^{{{\text{sf}}}}$ improvements of ~ 45% both at 4 K and 77 K with respect to the pristine values.

**Figure 4:**
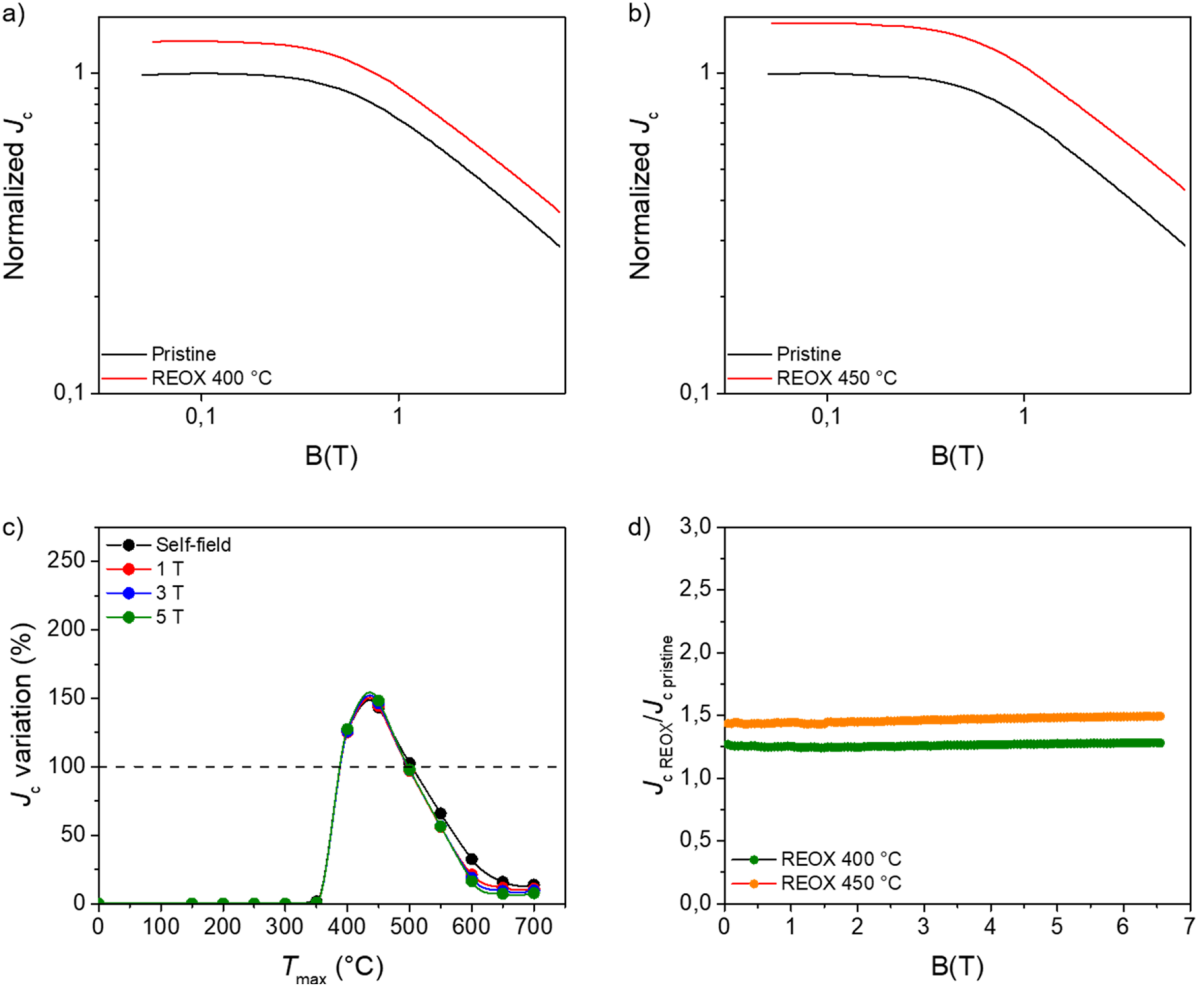
Normalized *J*_c_(B) curves at 4 K of (**a**) a pristine sample and the sample reoxygenated at 400 °C for 30 min; (**b**) a pristine sample and the sample reoxygenated at 450 °C for 30 min. The chart (**c**) displays the variation of *J*_c_ at 4 K at different magnetic fields in dependence of the reoxygenation temperature (*T*_reox_). The dark dashed line indicates the nominal values of the pristine sample. The solid lines are only a guide for the eyes. The graph (**d**) shows the ratio between the *J*_c_ of the reoxygenated samples (at 400 °C and 450 °C) and the pristine ones at 4 K in dependence of the magnetic field.

**Figure 5:**
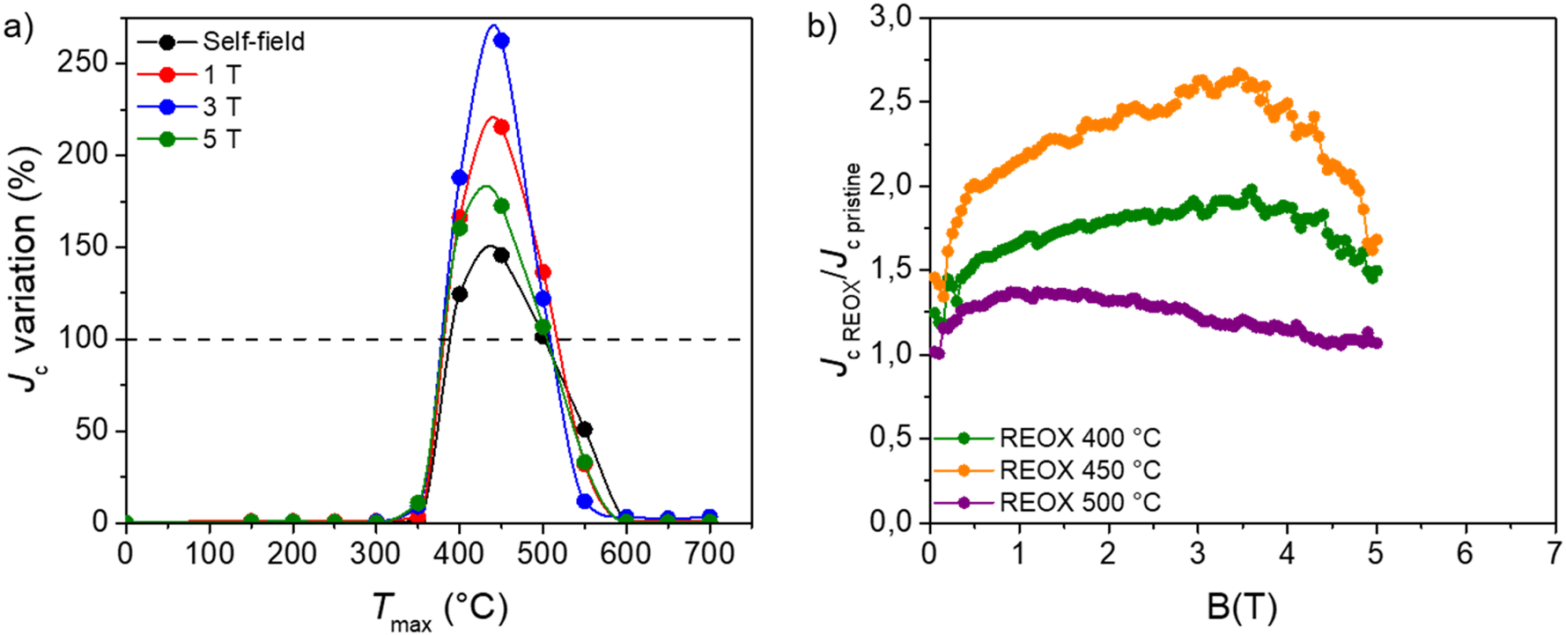
(**a**) The *J*_c_ variation at 77 K and different magnetic fields in dependence of the reoxygenation temperature (*T*_reox_). The dark dashed line indicates the nominal values of the pristine sample. The solid lines are only a guide for the eyes. (**b**) Ratio between the *J*_c_ of the reoxygenated (at 400 °C, 450 °C, and 500 °C) and the *J*_c_ of the pristine samples at 77 K in dependence of the magnetic field.

The observed improvement of the *J*_c_ values is not restricted to self-field, but also kept in field as we can observe in Figs. 4 and 5. Figure 4a, b show the *J*_c_(B) curves at 4 K of the pristine and reoxygenated samples at 400 °C and 450 °C (largest *J*_c_ increase). The curves were normalized to the *J*_c_ of the pristine sample at zero field. The graphs in Figs. 4c, d and 5a, b correspond to the ratio between the *J*_c_ values of the reoxygenated samples and the pristine ones at different fields.

At 4 K, we can observe in Fig. 4c, d that the maximum *J*_c_ increment, that happens when the samples are reoxygenated at 450 °C, is kept quite constant at ~ 45% in-field at least till 6.5 T, which is the maximum field of our magnetic measurements. Also, the increase of ~ 25% observed in the samples reoxygenated at 400 °C is maintained in field. The *J*_c_(B) curves in Fig. 4a, b show a power law dependence (*J*_c_ ~ B^−α^). The value of the exponent α is ~ 0.5 for the 4 curves.

At 77 K, we also observe a clear increase of *J*_c_ that extends in-field at least up to 5 T (above this field the signal is very noisy) for the samples reoxygenated at 400 and 450 °C. However, as it is observed in Fig. 5a, b and contrary to the case of 4 K, at 77 K the increment of *J*_c_ is not constant in-field. Figure 5b shows that for the reoxygenated samples with the largest increase of *J*_c_, i.e., the ones reoxygenated at 400 °C and 450 °C, the maximum enhancement is found at ~ 3.5 T. In particular *J*_c_ values are up to ~ 2.5 larger than the pristine ones in the case of the sample reoxygenated at 450 °C.

**Figure 6:**
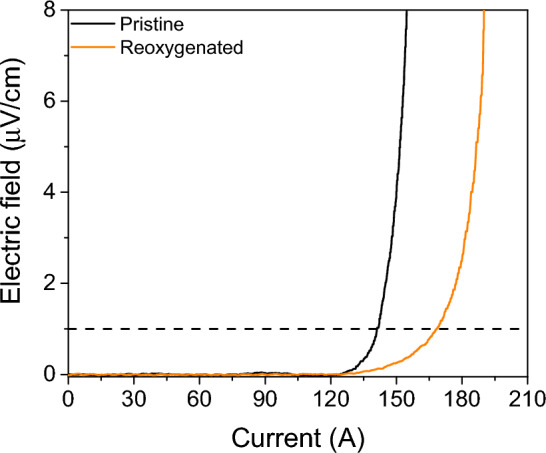
E–I curves at self-field and 77 K of a pristine sample compared with a sample reoxygenated at 400 °C during 30 min. The self-field *I*_c_ is determined by the crossing point of the black dashed line (1 µV/cm criterion) with both curves.

In order to confirm the observed improvement of *J*_c_ after reoxygenation in a more realistic case from the applications point of view, we have carried out a reoxygenation experiment in a 90 × 4 mm^2^ sample and then measured the self-field *I*_c_ ($I_{{\text{c}}}^{{{\text{sf}}}}$) at 77 K by transport. Also, as this experiment is viewed as an independent proof of the previously shown results, one particular set of conditions was selected: reoxygenation at 400 °C during 30 min (this sample was deoxygenated and reoxygenated following exactly the same process as for the small samples). The electric field vs. transport current (*E*–*I*) curves of the pristine sample and the one after reoxygenation are shown in Fig. 6. The $I_{{\text{c}}}^{{{\text{sf}}}}$ values at 77 K were 139.8 A for the pristine sample and 169.0 A for the reoxygenated one, which corresponds to an increase of ~ 21%. This increase is very similar to the one observed in the small sample measured by SQUID which reaches ~ 25% of improvement. This result confirms the validity of our experimental approach.

### Microstructural characterization by TEM

**Figure 7:**
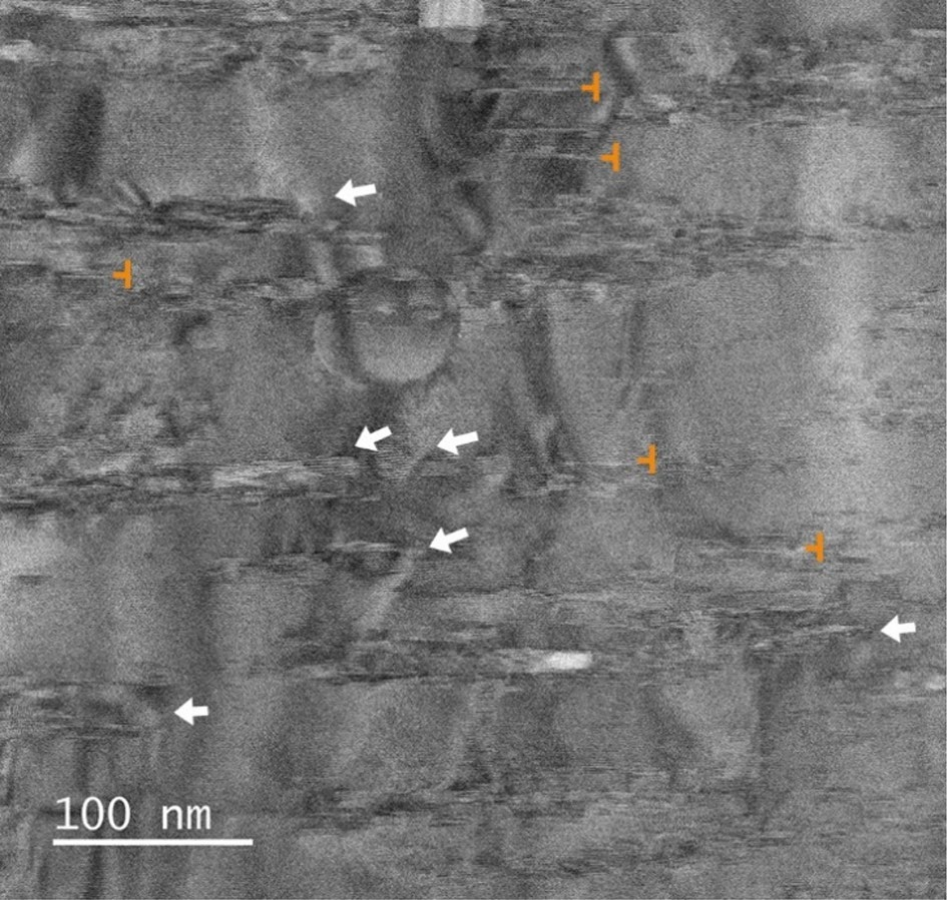
BF-STEM image of the matrix of YBCO film in a pristine sample. White arrows indicate Y_2_O_3_ nanoparticles and orange symbols stacking faults.

With the idea of gathering some microstructural information that helps to explain the results shown in the previous sections, we have carried out a TEM study on selected samples. Figure 7 presents a bright-field scanning transmission electron microscopy (BF-STEM) image of the YBCO matrix of a pristine sample. We can highlight two main features in this image. First, we can observe Y_2_O_3_ nanoparticles (NPs) embedded in the matrix (marked with white arrows in Fig. 7) that are formed naturally (they are not artificial pinning centers), as the manufacturer explained^1^, during the deposition and growth of the YBCO layer. Also, we can notice the presence of some rather short (20–50 nm) stacking faults (SFs, orange symbols in Fig. 7).

**Figure 8:**
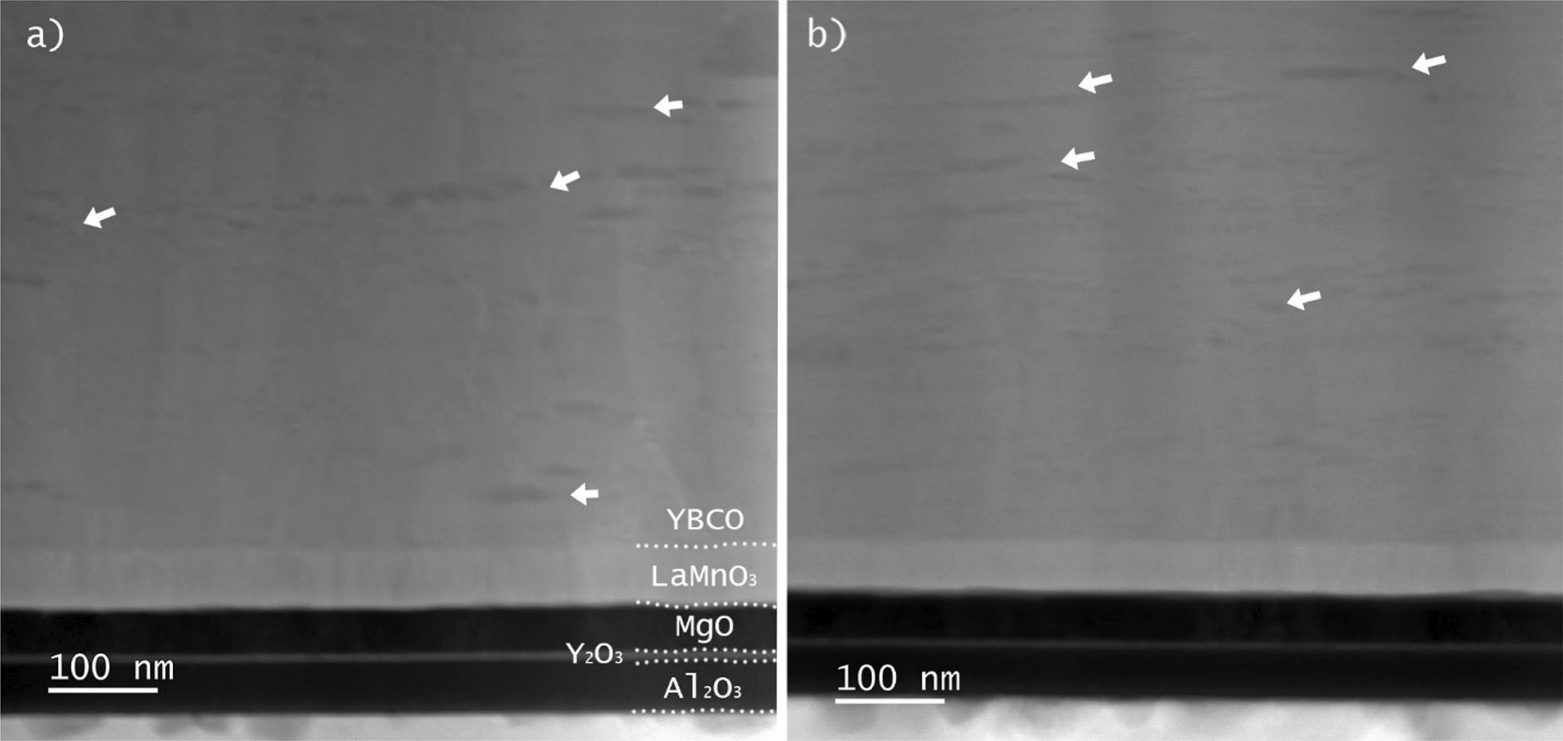
Cross-sectional HAADF-STEM image of the YBCO layer of (**a**) a pristine sample and (**b**) a sample reoxygenated at 600 °C during 300 min.

Looking at the high-angle annular dark field scanning transmission electron microscopy (HAADF-STEM) images shown in Fig. 8, we can compare the microstructure of a pristine sample (Fig. 8a) with a sample that underwent first a deoxygenation at *T*_deox_ = 700 °C and then a reoxygenation at *T*_reox_ = 600 °C during 300 min (Fig. 8b). We cannot perceive any big differences between both images. In both samples, we notice the presence of multiple black dots that are the Y_2_O_3_ NPs, marked with white arrows, and their distribution and density are not changing significantly after the reoxygenation. If this is the case of a sample reoxygenated at 600 °C during 300 min, one could expect that the same situation will be found at lower temperatures and shorter times.

## Discussion

The above presented results show that there is a significant effect of the deoxygenation/reoxygenation processes on the superconducting properties of a commercial coated conductor.

The deoxygenation experiments clearly reveal that the samples are not anymore superconducting when doing a deoxygenation process around 500–550 °C. At this temperature the transition from orthorhombic to tetragonal structure should occur. One interesting observation that deserves to be commented is the fact that while *T*_c_ remains constant, the $J_{{\text{c}}}^{{{\text{sf}}}}$ starts decreasing at very low temperatures with drops of ~ 3% at 150 °C and ~ 5% at 200 °C. This data confirms what was already reported and explained in a previous work of our group^22^. In summary, this reduction of $J_{{\text{c}}}^{{{\text{sf}}}}$ at low deoxygenation temperatures is attributed to the oxygen out-diffusion from the grain-boundaries, a process that has a lower activation energy than oxygen out-diffusion from the interior of the grains. When the oxygen leaves the grain boundaries, they become gradually weak-links that hinder the current flow. At higher temperatures, the activation energy of the oxygen out-diffusion from inside the grains is reached and then a sudden and more severe decay of $J_{{\text{c}}}^{{{\text{sf}}}}$ is observed.

In the case of the reoxygenation process, more results are worth to be discussed. The main one is undoubtedly the remarkable increase of *J*_c_, not only at self-field, but also in-field, that the samples show after a reoxygenation in the range of 400–450 °C both at 4 and 77 K. We also count with other two pieces of information. The first one is that the CC used in this work is extremely overdoped, i.e., with δ ≈ 0 in YBa_2_Cu_3_O_7-δ_. The particular CC that we examined in this study was developed for applications in the field of nuclear fusion, where the properties at low temperature and high field are the most demanded ones. One of the ways to achieve high performance at the target operating conditions for HTS fusion magnets, i.e. *T* = 20 K, *B* = 20 T^35^, is to increase the charge carrier (hole) density in the superconducting layer. It was shown in the literature that high hole doping levels lead to smaller anisotropy and higher irreversibility field and this translates into a larger critical current at high fields^36^. Furthermore, overdoping potentially improves grain connectivity, as grain boundaries tend to be underdoped with respect to the grains and, therefore, are closer to an optimal oxygen content in overdoped samples. The other important point is that, according to our TEM analysis, the amount, distribution or homogeneity of NPs or SFs do not vary in major way when a deoxygenation/reoxygenation cycle is carried out. It was especially relevant to find out no major differences in the SFs landscape because it is known that certain annealing processes under pure oxygen flow induce the formation of SFs^37^. This means that, in principle, we can discard the action of NPs or SFs as responsible for the increase of *J*_c_ that we found.

At 4 K, we observe an increase of *J*_c_ that could reach ~ 25% in samples reoxygenated at 400 °C and ~ 45% in samples reoxygenated at 450 °C. This improvement is constant in-field at least till 6.5 T. When analyzing the *J*_c_(B) curves of the pristine samples and those of the reoxygenated ones at 400 °C and 450 °C, we found α exponent values of ~ 0.5 for the four of them. This suggests that, even if there is an evident *J*_c_ increase, the pinning mechanism is not changing in the reoxygenated samples with respect to one of the pristine samples. The α ~ 0.5 has been reported at 4 K for samples differing in texture, thickness, and architecture^38^. This value is typically associated with the interaction between vortices nucleated at the grain boundaries and those strongly pinned to the dislocation cores^38–40^. Therefore, the observed increase of *J*_c_ should come from a change in the distribution, homogeneity or density of some type of defect compatible with this pinning mechanism. With the experimental data that we have, we are not able to give more in-depth explanations on this matter. Achieving a more complete understanding of the microstructural reasons responsible for the enhancement of *J*_c_ will be the subject of a future work. However, we can point out that twin boundaries are a type of defect that can potentially fit in this picture for the vortex pinning. The twin domain structure is potentially transformable by modifying the reoxygenation process. This means that the oxygenation process of the pristine samples can create a certain twin boundaries scenario that is likely different from the one that is formed after our deoxygenation/reoxygenation cycle. We have to consider that the oxygenation on the pristine samples is done on initially non-oxygenated samples. The successive phase transformations that the oxygen in- and out-diffusion produces may lead to a totally different twin domain structure in our reoxygenated samples. One plausible option is that, in this new twin-boundary landscape, the twin boundaries end up losing their initial vertical coherence. This would change their behavior from channeling planes, when they are vertically coherent, to pinning centers, specially at low temperatures, that impede vortex channeling^41^.

Regarding our study performed at 77 K, we observed again a significant enhancement of *J*_c_ in the samples reoxygenated at 400 °C and 450 °C and also a slight increase in the sample reoxygenated at 500 °C, especially in-field. However, in this case, as shown in Fig. 5a, b, the increase for each sample is not constant in the investigated range of fields, as in the case of 4 K, but changes reaching a maximum at ~ 3.5 T. This difference in the *J*_c_ should be related with the fact that both the vortex-defect and the vortex-vortex interactions are expected to evolve upon varying the temperature^38,42^. Furthermore, the rise of *T*_c_ that we observed in the samples reoxygenated between 400 °C and 500 °C should play a role on the measured *J*_c_, too. As we mentioned above, the CC investigated in this work is extremely overdoped, with a *T*_c_ in the range 88–89 K. After our reoxygenation process, we end up with samples that present *T*_c_ values of ~ 91–92 K, very close to what is expected for optimally doped YBCO. An increase of *T*_c_ by 3–4 K can have a significant impact on the values of *J*_c_ measured at 77 K. Indeed, it is known that a higher *T*_c_ causes an increase of the irreversibility field that entails a displacement of the irreversibility line. Therefore, there is a notable shorter distance between 77 K and *T*_c_ for the pristine samples than in the case of reoxygenated ones. Furthermore, we cannot exclude that the changes in the microstructure that we commented before and that have a significant impact at 4 K play a role at 77 K, too. However, on this matter one should specify that defects that are effective at 4 K are normally less effective at 77 K, and vice versa.

Based on this analysis, we can, first, rule out that the *J*_c_ improvement is caused by an ameliorated grain connectivity with respect to the pristine sample because pristine samples are overdoped and, thus, their grain boundaries should be more transparent. Discarding this option, we infer that the variations in the superconducting properties that we observed in the reoxygenated samples should come from a combination of the modifications in the doping level and in the microstructure (defects) that result from the deoxygenation/reoxygenation cycle. We emphasize that NPs or SFs should not be the main source of pinning, since the TEM images showed no major differences between the pristine and the reoxygenated sample. Furthermore, SFs are expected to be more effective as pinning centers when the magnetic field is applied perpendicular to the *c*-axis, which is not the geometry realized in our experiment. Other type of defects, e.g. twin boundaries, are more likely to be responsible of the observed variation of *J*_*c*_. To clarify this hypothesis, a much deeper and detailed TEM study would be needed. This is something that we are planning in the near future, too. Furthermore, it would be very interesting to repeat this type of experiments in commercial tapes with a different initial doping level, but not overdoped, in order to compare the recovery rate with the case of the overdoped CC.

Another interesting point to clarify is the fact that *J*_c_ increases with respect to the pristine one in samples reoxygenated at ~ 400–450 °C and decays quite fast after 450 °C while the *T*_c_ remains at ~ 91–92 K in the range 400–550 °C. One possible interpretation of this behavior could be related with the well-known “*T*_c_ parabola”^29^. As it was mentioned before, according to the phase diagram, the lower is the oxygenation temperature, the larger is the achievable oxygen content. This means that, in principle, in order to obtain the maximum oxygen content, we should carry out oxygenation processes at low temperatures. However, at low temperatures, mainly below 300 °C, the diffusion process is extremely slow and it is very likely that 30 min is not enough to achieve a significant oxygen content in the *RE*BCO structure. In our case, it seems that a reoxygenation at 400 °C is the point when the diffusion rate is large enough to obtain a significant oxygen content after 30 min. Nevertheless, if we continue increasing the reoxygenation temperature, according to the phase diagram, the oxygen content that is possible to achieve decreases. Therefore, the samples that are oxygenated at 500 °C have, in equilibrium, less oxygen than those at 400 or 450 °C. This lower oxygen content, combined with the changes in the microstructure that we commented before, should be the reason for the *J*_c_ decrease after 450 °C. The fact that these samples have similar *T*_c_ is a consequence of the “*T*_c_ parabola”. Samples with different oxygen content can be in opposite sides of the parabola, and thus have the same *T*_c_. In our case, it seems that the samples reoxygenated at 400 and 450 °C are very close to the optimally doped state but still in the overdoped side while the one reoxygenated at 500 °C is closer to the top of the parabola (optimally doped) and the one reoxygenated at 550 °C is in the underdoped side. In any case, changes of around 1 K in *T*_c_ are pretty small and, as we mentioned before, all these samples are very near to the optimally doped value.

It is important to note that these experiments can not be performed in Cu stabilized CCs. The outer Cu layer oxidizes during the heat treatment performed in O_2_ atmosphere and, more specifically, gets severely damaged at relatively low temperatures, around 500–600 °C. In other terms, the Cu stabilizer acts as a barrier against the in-diffusion of oxygen into the *RE*BCO layer.

## Conclusions

In this work we carried out a comprehensive and systematic study of the deoxygenation and reoxygenation processes in a commercial coated conductor. In the past, this topic was well-studied in superconducting films deposited on single crystal substrates but not on commercial tapes. We found out that a complete suppression of superconductivity occurs when deoxygenating the samples under pure argon at ~ 500–550 °C for 30 min. Samples completely deoxygenated recover and even exceed their pristine *T*_c_ and *J*_c_ values when doing a reoxygenation under pure oxygen flow at temperatures in the range 400–500 °C during only 30 min. The increase of *J*_c_, observed not only at self-field but also in-field and at both 4 and 77 K, is remarkably large, reaching a maximum when doing the reoxygenation at 450 °C. This improvement of *J*_c_ was confirmed by electrical transport experiments measuring the self-field *I*_c_ at 77 K of longer samples treated in the exact same way as the small ones. TEM analyses did not give evidence of clear variations in the presence of nanoparticles or stacking faults between the pristine and reoxygenated samples. Therefore, these types of defects should not be responsible of the observed enhancement of *J*_c_. Our hypothesis is that some of the reoxygenated samples present larger *J*_c_ values due to the combined action of a change in the microstructure and a different oxygen doping after the reoxygenation process. We suggest that a potential change in the twin domain structure may be the microstructural difference between pristine and reoxygenated samples, but this hypothesis should be supported by further and dedicated investigations that we are planning for a future work. The simple reoxygenation process employed in this work, that lead to a notable improvement of the *J*_c_, introduces a new set of parameters that can be used by manufacturers to optimize the performances of the CCs, specifically by selecting the appropriate *p*O_2_-temperature–time path for maximizing *J*_c_.

## Data Availability

All relevant data are included in the paper. Raw data are available from the corresponding authors upon request.
